# A fifty percent leucine-restricted diet reduces fat mass and improves glucose regulation

**DOI:** 10.1186/s12986-021-00564-1

**Published:** 2021-03-26

**Authors:** Ziheng Zhou, Hanrui Yin, Yajie Guo, Yuanyuan Fang, Feixiang Yuan, Shanghai Chen, Feifan Guo

**Affiliations:** 1 CAS Key Laboratory of Nutrition, Metabolism and Food Safety, Innovation Center for Intervention of Chronic Disease and Promotion of Health, Shanghai Institute of Nutrition and Health, University of Chinese Academy of Sciences, Chinese Academy of Sciences, 320 Yueyang Road, Shanghai, 200031 China; 2 Shanghai Jiao Tong University Affiliated Sixth People’s Hospital, Shanghai, China

**Keywords:** Obesity, Leucine restriction, Lipid metabolism pathway, Insulin signaling

## Abstract

**Background:**

Leucine deprivation modulates the dietary amino acid composition, reducing the fat content and improving the glucose tolerance, thus protecting the organism against obesity. However, a complete deprivation of leucine can lead to an extremely rapid fat loss in mice, accompanied by prolonged adverse effects such as weakness and mental fatigue. Therefore, in this study we aimed to seek the optimal concentration of dietary leucine that can reduce fat mass and improve the metabolism without the onset of severe effects.

**Methods:**

To investigate whether there is a better concentration of diet leucine restriction (LR), based on the diet we conducted (A10021B), that can reduce fat mass and improve metabolism status without taking many negative effects, we fed 8 weeks old male C57Bl/6J mice with increasing degrees of leucine restriction diet 0% LR (control group), 25% LR, 50% LR, and 75% LR groups (4–6 mice each group). Fat mass and blood glucose levels were measured. The expression levels of genes involved in lipid metabolism in white adipose tissue (WAT) and liver, and proteins in insulin signaling were assessed in WAT, liver and muscle.

**Results:**

We found that the 50% LR group is the most proper group here at the lowest leucine effective concentration, which reduced fat mass (*p* *< *0.05) and improved glucose regulation in mice over a 90 days feeding. Further studies revealed that lipid synthesis pathway (*Fas*, *Scd1and Srebp1*, *p* < 0*.*05) was downregulated and lipolysis (*Atgl*, *p* < 0*.*05) was upregulated in WAT in 50% LR group, compared to that in control group. Furthermore, glucose regulation (glucose tolerance test, *p* < 0.05) was also improved, and insulin signaling (*p* < 0.05) in the muscle was enhanced in 50% LR group while in WAT and liver were not changed.

**Conclusions:**

Collectively, a 50% LR in mice reduced fat mass and improved glucose regulation, which may function through modulating lipid synthesis and lipolysis pathway in adipose tissue as well as enhancing insulin signaling in muscle. So far, we provide a further consideration for carrying out the diet of leucine restriction to reduce fat and improve metabolism status before clinical study.

**Supplementary Information:**

The online version contains supplementary material available at 10.1186/s12986-021-00564-1.

## Background

Obesity is a global pandemic associated with an increased risk of developing type 2 diabetes, cancer, respiratory and cardiovascular diseases [1, 2]. Dietary restriction is a nutritional intervention with clear health benefits as it moderately reduces food intake, protects against multiple diseases, including obesity, cardiovascular disease, and diabetes, and extends life span in rodents [3]. Short-term diet restriction in humans also benefits glucose and energy homeostasis, increasing insulin sensitivity and reducing body fat [4]. Furthermore, many of the effects of dietary restriction on longevity and health span in model organisms have been linked to reduced protein and amino acid (AA) intake and the stimulation of specific nutrient signaling pathways [5, 6]. Protein or essential amino acid (EAA) restriction extends both lifespan and healthspan in rodents and reduces diabetes, cancer, and overall mortality in humans [7–9]. Thus, interventions aimed at lowering the intake of proteins or specific AAs can be beneficial and have the potential to be widely adopted and effective in preventing obesity and optimizing healthspan [9].

Clinical studies showed that circulating levels of branched-chain amino acids (BCAAs), which include three EAAs: leucine, isoleucine and valine, tend to be increased in individuals with obesity and are associated with worse metabolic health and future insulin resistance or type 2 diabetes mellitus [10]. A bunch of studies reported that conducting a restriction of one or more BCAAs in diet can improve body composition and glucose metabolism in lean and obese mice, as well as rats [11–13]. Previous studies found that a short-term (7 days) deprivation of dietary leucine, resulted in a reduction in food intake and body weight by modulating lipid metabolism in white adipose tissue (WAT) and liver in mice [14]. Further studies showed that leucine deprivation for 7 days in mice increased lipolysis in WAT and improved the whole-body insulin signaling [15, 16], leucine deprivation produces beneficial responses in short-term studies, however, consumption of one EAA complete deficient diet beyond 2–3 weeks rapidly jeopardizes the animal’s health [14, 15, 17].

Skeletal muscle is quantitatively the most important organ in maintaining glucose homeostasis and insulin sensitivity, which is responsible for approximately 80% of insulin-stimulated whole-body glucose uptake and disposal under normal conditions [18], and is a major organ of insulin resistance in type 2 diabetic patients [19]. Thus, skeletal muscle insulin pathway can be a potential target for improving glucose homeostasis and insulin sensitivity [20]. It has been reported that leucine deprivation in mice enhanced insulin signaling in muscle [16].

Since restriction of uptake of BCAAs, including leucine, can improve metabolic status, and total deprivation of one EAA of the diet has adverse effects for health in long term, the aim of our study is to seek for a manipulation in reduction of the uptake of leucine in diet while avoiding the potential negative effects in health. We speculated that whether there is a better concentration of dietary leucine that could reduce fat mass and improve metabolism without the onset of severe effects. To determine this speculation, we fed mice with leucine restriction (LR) diets: 0% LR (control diet) or with 25% LR, 50% LR, and 75% LR, examining body weight gain, fat mass and glucose regulation ability to evaluate the effects. We found that 50% LR is the better concentration here based on our diet composition that can lead to fat loss by downregulating lipid synthesis and upregulating lipolysis in WAT, and glucose tolerance improvement by enhancing insulin signaling in muscle.

## Methods

### Animals and diets

Wild-type male C57BL/6J mice were obtained from Shanghai Laboratory Animal Company (Shanghai, China). Eight- to ten-week-old mice were maintained on a 12 h light/dark cycle at 25 °C with free access to commercial rodent food and tap water before the experiments. 0% LR (Control diet, A10021B), 25% LR, 50% LR and 75% LR diets were obtained from Research Diets (New Brunswick, NJ). All diets were isocaloric and compositionally the same in terms of lipid, and caloric decreased in leucine was added in terms of carbohydrate (shown in the Additional file 1: Table S1).

**Fig. 1:**
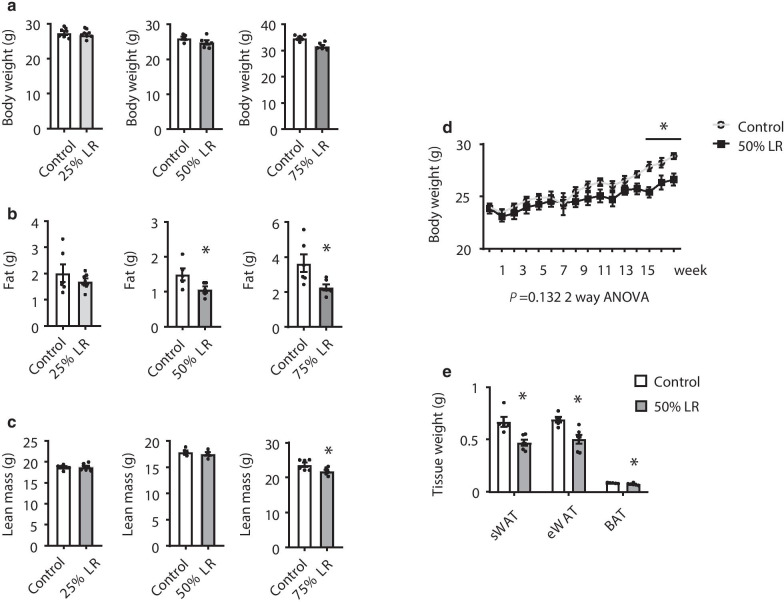
Leucine restriction (LR) has effect on fat mass reduction. **a**–**c** Body weight, fat mass, and lean mass of mice fed with 0% LR (control) or 25% LR (left), 50% LR (middle), and 75% LR (right) diet for 8 weeks. **d** Body weight of mice fed with LR (control) or 50% LR diet over 12 weeks. **e** Weight of subcutaneous white adipose tissue (sWAT), epididymal white adipose tissue (eWAT), and brown adipose tissue (BAT) of mice fed with 0% LR (control) or 50% LR diet. Data are expressed as the mean ± SEM (n = 4–6 per group, as indicated), with individual data points. *p < 0.05

For Fig. 1a, b, at the start of the feeding experiment, mice were acclimated to a control diet for 3 days and then randomly divided into paired groups, each paired group accepted two treatments, either 0% LR (control group) or one of 25% LR, 50% LR, and 75% LR, and received their diets for 6 weeks (4–6 mice per group). Beside this, in the following experiments, mice were divided into control and 50% LR groups randomly, and received their diets over 3 months. Weekly food intake, body weight, and body composition were measured. Lipid metabolism pathway was assessed in WAT and liver. In addition, glucose tolerance tests (GTTs) and insulin tolerance tests (ITTs) were performed.

Mice were euthanized by decapitation following CO_2_-induced narcosis. Trunk blood was collected and processed for serum. Adipose tissues (WAT and brown adipose tissue), liver and muscle were isolated and immediately placed in 4% paraformaldehyde buffer for histological studies, or snap-frozen and stored at − 80 °C for future analysis. These experiments were conducted in accordance with the guidelines of the Institutional Animal Care and Use Committee of Shanghai Institutes for Nutrition and Health, Chinese Academy of Sciences.

### Body composition measurements

Mouse body composition was determined using a nuclear magnetic resonance system (Bruker, DE, USA). The mouse was placed into a tube and contained with a second tube to allow for only small movements of the mouse and accurate scanning. After measurement finished, put the mice back to the cage.

### Indirect calorimetry experiment

Mice were maintained in a comprehensive lab animal monitoring system (Columbus Instruments, Columbus, OH) for 24 h, according to the manufacturer’s instructions. The O_2_ consumption and CO_2_ production levels were continuously recorded over a 24 h period.

### Rectal temperature measurement

The rectal temperatures of the mice were measured during 9:00–17:00 in light cycle using a rectal probe attached to a digital thermometer (Physitemp. Inc., NJ, USA).

### Blood glucose, serum insulin, GTT and ITT

For measuring blood glucose, the blood levels were measured in fed (ad libitum fed,) or fasting (overnight fasting in GTT; 4 h fasting in ITT) with blood obtained from the tail tip. For measuring serum insulin levels, the blood samples were obtained in fed (ad libitum fed) or fasting (overnight fasting) from the tail vein bleeding and then centrifuged to collect the serum. Blood glucose levels were measured using a Glucometer Elite monitor. Serum insulin levels were measured using the Mercodia Ultrasensitive Rat Insulin ELISA kit (ALPCO Diagnostic, Salem, NH). GTTs and ITTs were performed by intraperitoneal injection of 2 g/kg glucose after overnight fasting and 0.75 units/kg insulin after 4 h of fasting, respectively.

### Serum amino acids concentration

The blood samples were obtained in fed from heart blood and then centrifuged to collect the serum. Serum was separated with centrifugation at 3500*g* for 25 min at 4℃ and stored at − 80℃. Serum amino acids were analyzed with high-performance liquid chromatography (Ultimate 3000, USA)-tandem mass spectrometry (API 3200 Q-TRAP, USA) methods by Beijing MS Medical Research Co. Ltd (Beijing, China).

### In vivo insulin signaling assay

Mice maintained on different diets were fasted for 6 h before insulin injection as previously described [21]. Sections of liver, soleus muscle, and abdominal fat were excised from anesthetized mice and snap-frozen, as untreated controls. Three to five minutes after injection with 2 units/kg of insulin via the portal vein, pieces of tissue section were excised and snap-frozen for Western blot analysis.

### Western blot analysis

Whole-cell lysates from frozen tissues were isolated using RIPA lysis buffer (150 mM Tris–HCl, 50 mM NaCl, 1% NP-40, and 0.1% tween-20). Protease and phosphatase inhibitors were added to all buffers before experiments. Western blot was performed as previously described [15, 16]. Protein concentrations were assayed with BCA Kit (Pierce). The information of antibodies was appended in Additional file 1: Table S2. Primary antibodieswere incubated overnight at 4 °C and specific proteins were visualized by ECL Plus (Beyotime Biotechnology). Band intensities were measured using Quantity One (Bio-Rad Laboratories) and normalized to that of actin or non-specific proteins.

### RNA isolation and relative quantitative reverse transcription PCR (RT-PCR)

RT-PCRs were performed as previously described [15]. Total RNA was prepared from frozen tissues with TRIZOL (Invitrogen) reagent. One microgram of RNA was reverse transcribed with PrimeScript RT reagent kit (Takara). Quantitative amplification by PCR was carried out using SYBR Green I Master Mix reagent with an ABI 7500 system (Applied Biosystem). PCR products were subjected to a melting curve analysis. Cycle numbers of both *Gapdh* (as an internal control) and cDNAs of interest at a specific threshold within the exponential amplification range were used to calculate relative expression levels of the genes of interest. The sequences of primers used in this study are appended in Additional file 1: Table S3.

#### Statistical analysis

All data are expressed as mean ± standard error of the mean (SEM). Significant differences between groups were assessed using the two-tailed Student’s t-test. For body weight curve, GTTs and ITTs, data were analyzed for statistical significance using Student’s *t*-test to compare the difference between different groups of mice at each time point examined; testing multiple time points of the curve was analyzed using two-way repeated measures ANOVA. *p*-value < 0.05 was considered statistically significant.

## Results

### A 50% LR reduces fat mass

To determine the optimal concentration of dietary leucine that could reduce fat mass and improve metabolism status, we fed mice with four different LR diets: 0% LR, 25% LR, 50% LR, and 75% LR. None of the groups evidenced a significant change in body weight after 8 weeks of feeding, compared to the control group (Fig. 1a). As previously reported that leucine deprivation can induce fat loss in mice, we found similar results that fat mass reduced significantly in both 75% LR and 50% LR groups, but not in 25% LR group (Fig. 1b). Besides, mice in the 75% LR exhibited a significantly reduction of lean mass, which may cause side-effects (Fig. 1c). While 50% LR did not influence lean mass (Fig. 1c). Other tissue weights were not changed between two groups (Additional file 1: Fig. S1A), while the fluids were increased in 50% LR compared to control diet (Additional file 1: Fig. S1B). Based on that 50% LR can decrease fat mass without obvious influence on tissues weights, we chose the 50% LR diet as the proper concentration for the following experiments.

Mice fed with 50% LR decreased the weight of body and adipose tissue including subcutaneous white adipose tissue (sWAT), epididymal white adipose tissue (eWAT), and brown adipose tissue (BAT) significantly over 90 days (Fig. 1d, e).

### A 50% LR does not change serum leucine concentration

Previous study showed that leucine facilitates both tissue uptake of branched-chain amino acids and their intracellular metabolism [22]. It has been reported that leucine deprivation results in a decrease in leucine and increase in isoleucine, valine, and several other AA levels in the serum of mice [16]. Therefore, we measured the AA concentration in serum. Consistently, the levels of the other two branched chain AAs were increased (Additional file 1: Fig. S2). Intriguingly, leucine almost did not decrease in mice fed with 50% LR diet (Additional file 1: Fig. S2), suggesting that the blood level of leucine is regulated post-ingestively.

**Fig. 2:**
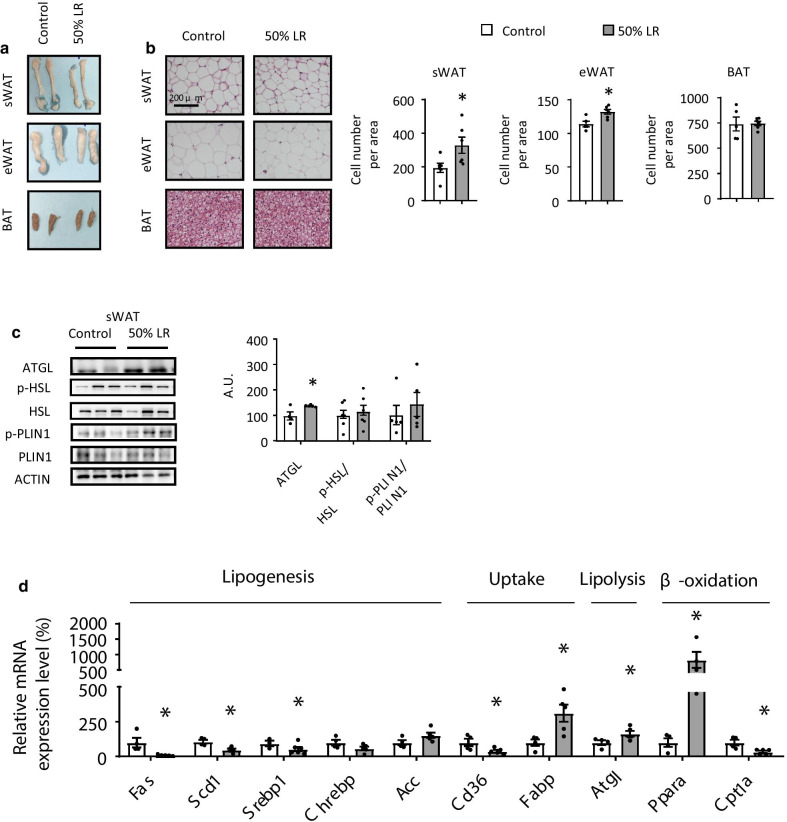
A 50% leucine restriction (LR) reduces fat mass by mainly inhibiting lipogenesis. **a** Direct images of subcutaneous white adipose tissue (sWAT), epididymal white adipose tissue (eWAT), and brown adipose tissue (BAT). **b** Hematoxylin and eosin (H&E) staining of adipocytes in sWAT (top), eWAT (middle), and BAT (bottom). **c** Western blotting and quantification of the protein levels of p-HSL, HSL, p-PLIN1, PLIN1 and ATGL in sWAT in 0% LR (control) or 50% LR. A.U.: arbitrary unit. **d** Relative mRNA expression levels of genes involved in lipogenesis, uptake, lipolysis and β-oxidation were measured. All mice were fed with a 0% LR (control) or 50% LR diet. Data are expressed as the mean ± SEM (n = 4–6 per group, as indicated), with individual data points. *p < 0.05

### Lipid synthesis is downregulated and lipolysis is upregulated in WAT

To determine the cause behind the reduction in adipose tissue of mice from the 50% LR group (Fig. 2a), we conducted a hematoxylin and eosin (H&E) staining. Our results revealed that the adipocytes of both sWAT and eWAT in 50% LR group were smaller in size than those in the control group and the cell number per area was increased, but not of BAT (Fig. 2b). The reduced size of adipocytes in mice fed with 50% LR could be due to an enhanced lipolysis or reduced lipid synthesis [15]. Protein level of phosphorylation of hormone sensitive lipase (p-HSL), the rate-limiting enzyme for triglyceride lipolysis [23], and phosphorylation of perilipin 1 (PLIN1), which is also important for lipolysis [24], were not changed between 50% LR and control group (Fig. 2c). While the adipose triglyceride lipase (ATGL) was significantly increased in 50% LR compared to that in control diet (Fig. 2c). Furthermore, genes involved in lipogenesis, including *Srebp1c* (sterol regulatory element-binding protein 1c), *Chrebp* (carbohydrate-responsive element-binding protein), *Acc* (acetyl-CoA carboxylase), *Fas* (fatty acid synthase), and *Scd1 *(stearoyl-CoA desaturase); gene involved in lipolysis, including *Atgl*; genes involved in fatty acid oxidation, including *Ppar*α (peroxisome proliferator-activated receptor α), and *Cpt1*α (carnitine palmitoyl transferase 1a); and genes involved in fatty acid uptake, including *Cd36 *(cluster of differentiation 36), and *Fabp *(fatty acid binding protein), were examined by RT-PCR [25]. The levels of some lipogenesis genes such as *Fas*, *Scd1,* and *Srebp1c*, were significantly decreased in 50% LR group. The expression of key genes involved in β-oxidation and lipolysis, *Pparα* and *Atgl* were enhanced approximately by 8- and 1.5-fold, respectively, in 50% LR group, compared to that in control group (Fig. 2d). In addition, the crucial receptor for fatty acid uptake, *Cd36,* was decreased significantly in 50% LR group (Fig. 2d). While lipids metabolism in liver was not changed between two groups (Additional file 1: Fig. S3). These results above suggested that decreased lipogenesis and enhanced lipolysis might primarily contribute in some extent to fat loss in 50% LR group.

**Fig. 3:**
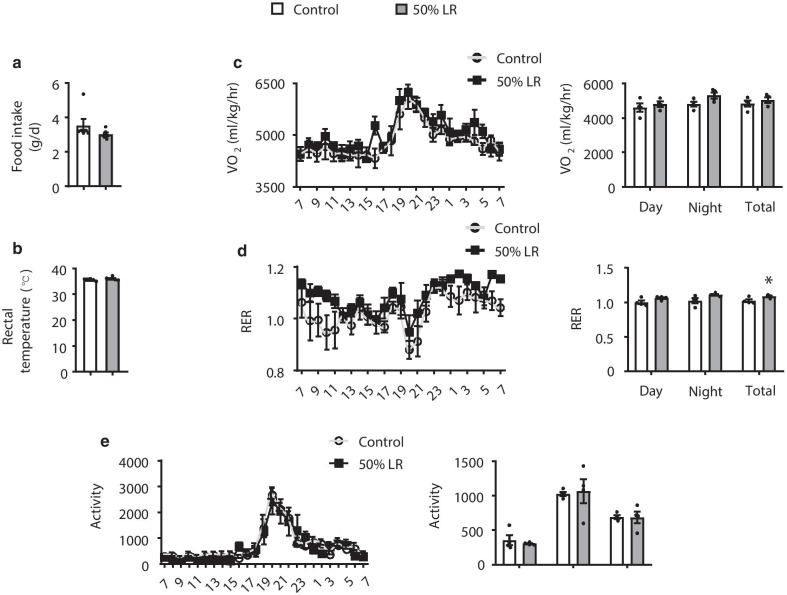
A 50% leucine restriction (LR) does not interfere with energy balance. **a** Daily food intake. **b** Rectal temperature. **c**–**e**. 24 h oxygen consumption normalized by lean mass (**c**), respiratory exchange ratio (VCO_2_/VO_2_) (**d**), and locomotor activity (**e**). All mice were fed with a 0% LR (control) or 50% LR diet. Data are expressed as the mean ± SEM (n = 4–6 per group, as indicated), with individual data points. *p < 0.05

### A 50% LR does not change energy homeostasis

Energy homeostasis is maintained by a balance between food intake and energy expenditure [26]. Therefore, an enhanced lipolysis suggested that the energy balance was disrupted. However, food intake (Fig. 3a) and the rectal temperature (Fig. 3b) in mice from the 50% LR group did not change. Furthermore, although total respiratory exchange ratio (RER; VCO_2_/VO_2_) increased (Fig. 3d), oxygen consumption and locomotor activity did not change in 50% LR group (Fig. 3c, e). Therefore, we excluded the possibility that fat loss was caused by a decrease in energy requirement or increase in energy usage.

### Glucose tolerance and insulin sensitivity are improved in 50% LR group with enhanced insulin signaling in muscle

**Fig. 4:**
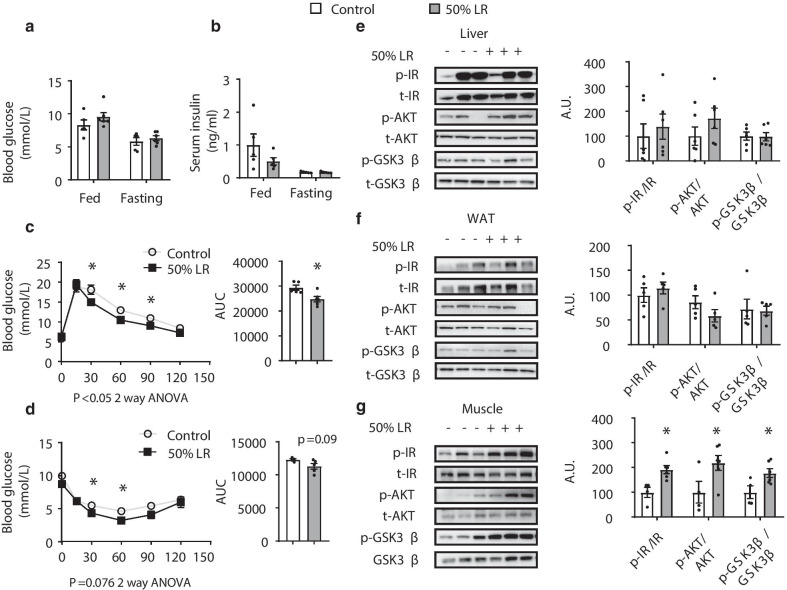
A 50% leucine restriction (LR) improves glucose regulation by enhancing insulin signaling in muscle. **a** Fed and fasting blood glucose levels. **b** Fed and fasting serum insulin levels. **c** Glucose tolerance tests (GTTs). **d** Insulin tolerance tests (ITTs). **e**–**g** Western blotting and quantification of the protein levels of P-IR, t-IR, p-AKT, t-AKT, p-GSK3β, and t-GSK3β in liver (**e**), white adipose tissue (WAT) (**f**), and muscle (**g**). A.U.: arbitrary units. All mice were fed with a 0% LR (control) or 50% LR diet. Data are expressed as the mean ± SEM (n = 4–6 per group, as indicated), with individual data points. *p < 0.05

Previous studies showed that leucine deprivation can improve glucose tolerance and insulin sensitivity in mice [16]. It showed that there were no differences between two groups in fed or fasting glucose or insulin levels (Fig. 4a, b). GTT indicated that 50% LR improved the mouse performance in modulating glucose levels (Fig. 4c). ITT indicated that insulin sensitivity was improved under a 50% LR (Fig. 4d). Then we checked the insulin signaling pathway in liver, WAT, and muscle. Interestingly, the levels of phospho-IR, phospho-AKT, and phospho-GSK3β proteins, involved in insulin signaling, were significantly enhanced only in muscle in 50% LR group; meanwhile they were unchanged in either liver or WAT (Fig. 4e–g). All the above results indicated that a 50% LR diet might improve glucose tolerance and insulin sensitivity by enhancing insulin signaling in muscle.

### A 50% LR diet does not reduce fat mass and improve fed blood glucose level of leptin receptor-deficient (db/db) mice

**Fig. 5:**
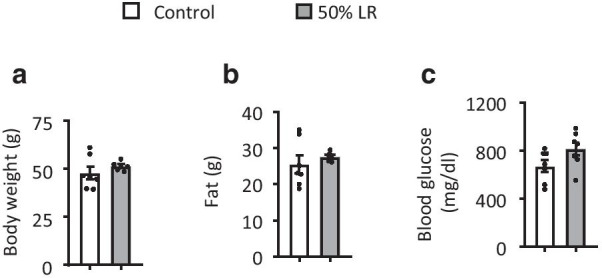
A 50% leucine restriction does not improve blood glucose levels in db/db mice. **a**. Body weight of db/db mice fed with a 0% LR (control) or 50% LR diet for 6 weeks. **b** Fat mass. **c** Fed blood glucose levels. All mice were fed with a 0% LR (control) or 50% LR diet. Data are expressed as the mean ± SEM (n = 7 per group, as indicated), with individual data points. *p < 0.05

Db/db mice are one of the most common animal models of studying type 2 diabetes due to their obesity and high level of glucose [27]. Therefore, we fed db/db mice with 50% LR diet to investigate whether a restrictive diet would ameliorate the metabolic syndrome. Body weight and fat mass did not change in 50% LR group (Fig. 5a, b). The fed blood glucose in 50% LR had no significant difference compared to the control group (Fig. 5c).

## Discussion

Many studies have proved that dietary composition plays an important role in overall health [28, 29]. Special attention has been focused on how dietary protein concentration and composition affect the energy balance and lifespan [5, 30]. However, the mechanism that how the body senses and responds to the changes in the dietary protein, and thus how the metabolic status can be improved by modulating dietary protein composition are still poorly understood.

Previous study has shown that full deprivation of dietary leucine can decrease fat mass and improve glucose tolerance in mice [16]. However, mice fed long term without leucine dramatically lost their body weight in a short time and had some adverse effects [14, 17]. Therefore, we supposed that if there is better degree of LR that could not only reduce fat mass but also avoid adverse effects. In this study, we observed that a 50% LR diet led to fat mass loss by decreasing the volume of adipocytes. Glucose tolerance was improved, the upregulation of insulin signaling in muscle tissue may have contribution to it in some extent.

To determine the threshold of dietary leucine required for activating the sensing signaling of the body to reduce fat based on the diet composition we used here, we investigated three different degrees of LR. 50% and 75% LR can both significantly reduce fat mass in mice, but only the 50% LR did not affect other body compositions. Therefore, we chose 50% LR diet as the best group to study the mechanism by which leucine restriction can lead to fat loss. Similar to full deprivation of leucine, we noted that 50% LR significantly reduced mouse body weight and fat mass over three months feeding, which was also observed in a recent study with the threshold of 80% LR [31]. The difference between the two studies relies in the diet composition: in our case, the deprivation of energy from administering a reduced concentration of leucine to the experimental groups was supplemented by the increase in energy levels derived from other carbohydrates added to the diet, without changing any other AAs. The other group added other AAs to complement the energy loss due to the reduced levels of leucine contained in the diet, without changing carbohydrates or fat. That’s complicated since we cannot exclude how much contribution other amino acids made under this situation. As recently reported that changing branched chain amino acids, including leucine, can modulate energy metabolism via a mechanism that involves their interaction with tryptophan and threonine [32]. Lees’ study also showed that leucine restriction diet (80% LR) has effects for weight and fat mass decrease, and glucose regulation improvement. Similar to us, they found no differences of 80% LR compared to control diet in hepatic lipogenesis, WAT and hepatic insulin signaling [33]. However, food intake and lipogenesis pathway in WAT were increased significantly in 80% LR compared to control group, which is different to us. We speculated that the increased food intake and enhanced lipogenesis may be a way of the mice for compensating the quick reduction of body weight.

As shown in previous study, leucine deprivation can reduce fat mass by enhancing lipolysis in WAT [15]. In this study, the cell volume of adipocytes in mice fed with 50% LR was smaller than that in the control group in sWAT. Intriguingly, the expression of mRNA and protein level of ATGL, which is important for lipolysis was upregulated, whereas that of many genes involved in lipid synthesis (*Fas*, *Scd1* and *Srebp1*) was significantly downregulated in 50% LR group. Thus, this decrement of lipogenesis process may be explained by the fact that leucine accounts for as much as about 30% of lipogenic acetyl-coenzyme A pools in adipocytes [34]. These data indicated that a 50% LR diet reduced fat mass by inhibiting fatty acid synthesis. Fontana et al. reported that leucine restriction (67%) actually increases the size of several fat depots including eWAT and does not decrease overall fat mass [11], however, the diet composition of us is quite different from that of Fontana’s, which may help explain the different results between us. And as Fontana conjectured, that the phenotypes they observed could result from an imbalance between the levels of leucine and either isoleucine or valine.

Adipose tissue plays an important role in regulating carbohydrate and lipid homeostasis. The scale of energy balance comprises energy intake and energy expenditure, each of which influences the mass of adipose tissue [35]. Firstly, we measured daily food intake between two groups, as a previous report showed that leucine deprivation reduced food intake [14]. No difference was found between two groups. We speculated that if the energy expenditure was increased as previously reported [15]. However, the rectal temperature was similar between two groups. The metabolic cage showed that except the total RER in 50% LR was significantly higher than that in the control group, no significant difference was found in oxygen consumption or activity between two groups. Above all, it showed that 50% LR did not change the energy balance, and the reduction of fat mass may have resulted directly from the lack of a source for lipogenesis.

Though leucine deprivation reduces food intake and decreases fat mass, several studies found that leucine supplementation can also have these effects [36]. However, whether leucine supplementation reduces food intake depends on the way it given. Studies evaluating the effects of central leucine infusion [37–39] and the effects of leucine supplementation in the diet reported decreased food intake [38–41]. No changes in food intake were observed in mice that received leucine supplementation through gavage, intraperitoneal injection or subcutaneous injections [36]. The effect of leucine supplementation favoring adiposity reduction may act directly by affecting the FAS expression and lipid catabolism on the adipocyte [42]. Besides, in vitro study demonstrates that leucine promotes energy partitioning from adipocytes to skeletal myotubes in co-culture systems, leading to net reductions in adipocyte lipid storage and increases in muscle fatty acid oxidation [43]. Thus, the effects of leucine restriction or supplementation on improvement of metabolic diseases depend on its subtle biological concentration.

Leucine deficiency can improve glucose tolerance in mice by increasing insulin sensitivity [16]. Fed and fasting blood glucose levels did not change between two groups. While GTT and ITT results showed that the glucose regulation in mice fed with a 50% LR diet was improved. Next, we speculated that which tissue contributes to the whole body insulin signaling in this condition. Serum insulin level was measured and no difference between two groups was noted. Levels of key proteins involved in insulin signaling, such as phospho-IR, phospho-AKT, and phospho-GSK3β, remained unchanged in WAT and liver, but were significantly enhanced in muscle. As leucine is an important AA for muscle metabolism and function maintenance [44–46], and can modulate insulin signaling in muscle under some situations [47, 48], we conjectured that 50% LR induced a specific signal that could exactly induce insulin signaling improvement in muscle, but not in liver or WAT. And the enhanced insulin signaling in muscle is enough to improve the whole body glucose regulation [20, 33].

The original aim of this study was to find a therapeutic approach to improve obesity or diabetes treatment. Therefore, we investigated the effect of this diet in db/db mice, which is generally thought as a genetic model of high glucose level and overweight [27], to determine whether LR could reduce fat or improve glucose levels. Results of fat mass and fed blood glucose level showed that no changes were observed between two groups. Some studies have reported that leucine has a synergetic effect with leptin, part of function of leucine requires a complete leptin signal pathway, thus complete deprivation of leucine did not improve in db/db mice, compared to that of the control diet [49, 50], which may explain, somehow, why 50% LR did not change fat and fed blood glucose level. However, we cannot exclude the possibility that 50% LR may have some beneficial effects in glucose regulation as we did not conduct GTT or ITT experiments.

Several limitations of our study are acknowledged. First, although the serum leucine concentration in 50% LR group was similar to that in control, we did not measure the concentration of leucine directly in adipose tissue. As adipose tissue is one of the important depots for metabolizing quantities of leucine [51], further studies should determine whether the uptake of leucine from circulation, protein turnover or AA oxidation differs in adipose tissue between two groups as previously reported [52].

In addition, why insulin signaling was enhanced only in muscle, and how muscle responded to the 50% LR diet still needs to be uncovered. If this response was direct, in vitro studies with muscle cell lines could provide more insights*.*

In the last, db/db mice can not represent the obesity model of high fat feeding. High fat feeding induced obesity may mimic the circumstances that high energy intake lifestyle in human life in nowadays, the effect of leucine restriction under this model need to be studied further.

## Conclusions

Above all, we singled out a better degree of LR to reduce body fat and improve glucose regulation in mice. A 50% LR significantly decreased fat mass by decreasing lipid synthesis in WAT, and improved glucose tolerance by increasing insulin sensitivity in muscle. One step closer to clinical therapy of appropriate dietary leucine deficiency in order to treat obesity and diabetes was investigated here. However, further studies focusing on how insulin signaling is activated in muscle are supposed to be followed.

## Supplementary Information

**Additional file 1.** More details of Weight of tissues (Additional Fig S1), Serum amino acids concentration (Additional Fig S2), Liver lipid metabolism (Additional Fig S3), Diet composition of all diets (Additional Table S1), Antibodies information (Additional Table S2) and RT-PCR primer sequences (Additional Table S3).

## Data Availability

All data, analytic methods, and study materials presented within this article and in the Data Supplement are available for other investigators from the corresponding authors on reasonable request.

## Abbreviations

LR: Leucine restriction
AA: Amino acid
EAA: Essential amino acids
WAT: White adipose tissue
SEM: Standard error of the mean
eWAT: Epididymal white adipose tissue
BAT: Brown adipose tissue
sWAT: Subcutaneous white adipose tissue
db/db: Leptin receptor-deficient
RER: Respiratory exchange ratio
