# Supplementary material for: A fifty percent leucine-restricted diet reduces fat mass and improves glucose regulation
Source: Nutr Metab (Lond). 2021 Mar 26;18:34. doi: 10.1186/s12986-021-00564-1 (PMC7995702; doi:10.1186/s12986-021-00564-1)
Supplement: Supplementary file 1 — Additional file 1. More details of Weight of tissues (Additional Fig S1), Serum amino acids concentration (Additional Fig S2), Liver lipid metabolism (Additional Fig S3), Diet composition of all diets (Additional Table S1), Antibodies information (Additional Table S2) and RT-PCR primer sequences (Additional Table S3). [file 12986_2021_564_MOESM1_ESM.doc]

**Supplementary Figures and Tables**


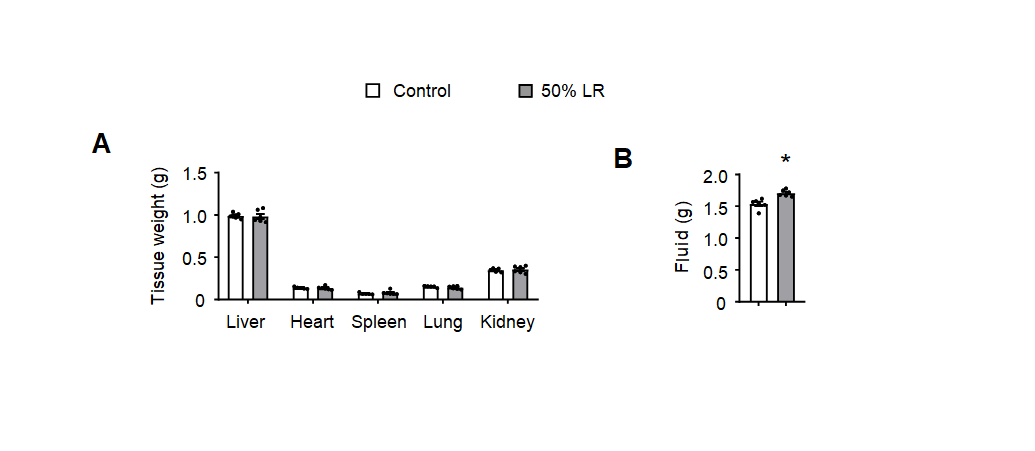


**Supplementary Figure 1. Weight of tissues and fluid of control and 50% leucine restriction (50% LR) mice**. A. Tissue weight of liver, heart, spleen, lung and kidney in control diet and 50% LR. B. Weight of Fluid in control diet or 50% LR. All mice were fed with a 0% LR (control) or 50% LR diet. Data are expressed as the mean ± SEM (n = 5-6 per group, as indicated), with individual data points. *p<0.05.


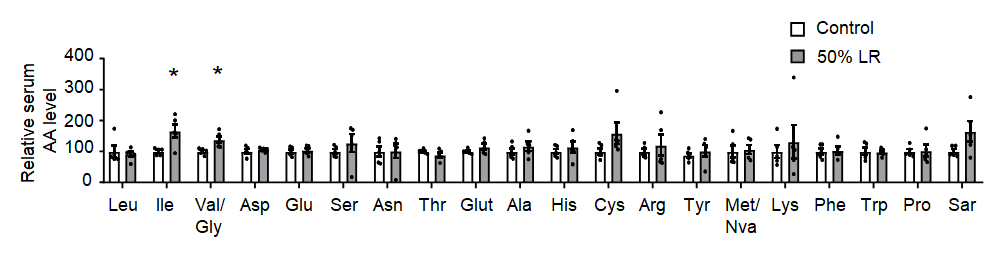


**Supplementary Figure 2.** **50% LR does not change serum leucine concentration.** Relative serum amino acids level in control and 50% LR. Leu: leucine, Ile: isoleucine, Val/Gly: valine/glycine, Asp: asparagine, Glu: glutamate, Ser: serine, Asn: Asparagine, Thr: threonine, Glut: Glutarnine, Ala: alanine, His: histidine, Cys: cystine, Arg: arginine, Tyr: tyrosine, Met: methionine, Lys: lysine, Phe: phenylalanine, Trp: trptophan, Pro: proline, Sar: sarcosine. All mice were fed with control or 50% LR diets. Data are expressed as the mean ± SEM (n = 5 per group, as indicated), with individual data points. **p* < 0.05.


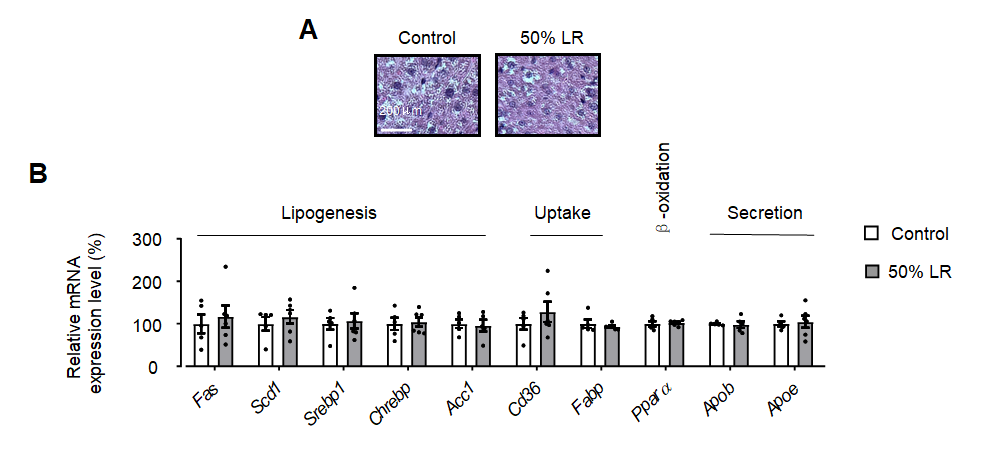


**Supplementary Figure 3. Lipid metabolism was not changed in 50% leucine restriction (50% LR) group compared to control group**. A. Hematoxylin and eosin (H&E) staining of of liver section of control and 50% LR. B. Relative mRNA expression level of genes involved in lipid metabolism pathway in liver. All mice were fed with a 0% LR (control) or 50% LR diet. Data are expressed as the mean ± SEM (n = 5-6 per group, as indicated), with individual data points. *p<0.05.

**Supplementary Table 1**

**Diet composition of all dietary treatments**.


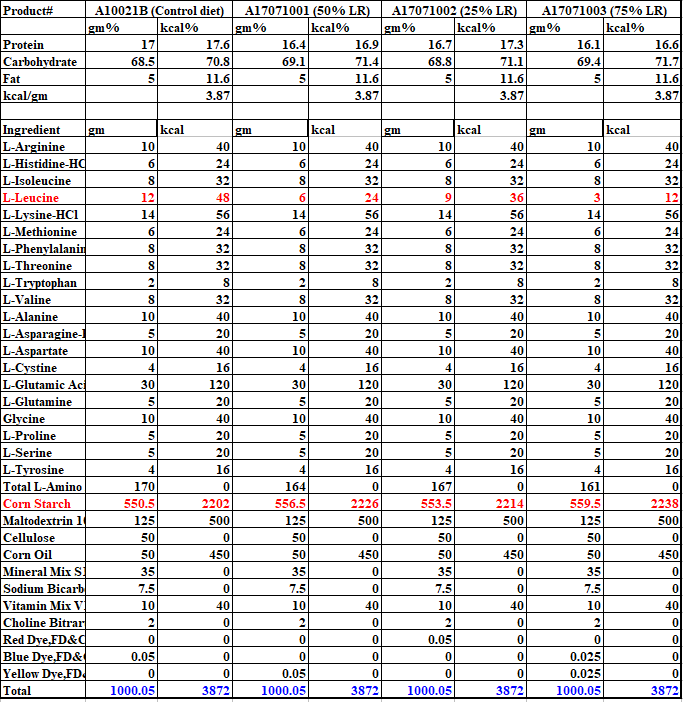


**Supplementary Table 2**

**Antibodies information**.

| **Antibodies** | **Source** | **Cat number** |
| --- | --- | --- |
| P-HSL (Ser660) | Cell Signaling Technology | 4126S |
| HSL | Cell Signaling Technology | 4107S |
| P-IR | Cell Signaling Technology | 3024s |
| IR | Cell Signaling Technology | 3025S |
| p-AKT(Ser473) | Cell Signaling Technology | 9271S |
| Akt | Cell Signaling Technology | 9272S |
| P-GSK3beta(Ser9) | Cell Signaling Technology | 9336s |
| GSK3beta(27C10) | Cell Signaling Technology | 9315s |
| p-Perilipin | Vala Science | 4855 |
| Perilipin | Vala Science | 4854 |
| ATGL | Abclonal | A6245 |
| ACTIN | ProteinTech | 66009-1-Ig |
| Goat-anti mouse | Jackson | 115-035-003 |
| Goat-anti rabbit | Jackson | 111-035-003 |

**Supplementary table 3**

**Primers for quantitative RT-PCR**

| **Primer** | **Sequence** |
| --- | --- |
| *Acc-*F | 5′-TGACAGACTGATCGCAGAGAAAG-3′ |
| *Acc-*R | 5′-TGGAGAGCCCCACACACA-3′ |
| *Atgl-*F | 5'-GTGAAGCAGGTGCCAACATTATTG-3' |
| *Atgl-*R | 5'-AAACACGAGTCAGGGAGATGCC-3' |
| *Cd36-*F | 5′-TGGAGCTGTTATTGGTGCAG-3′ |
| *Cd36-*R | 5′-TGGGTTTTGCACATCAAAGA-3′ |
| *Chrebp-*F | 5′-CTGGGGACCTAAACAGGAGC-3′ |
| *Chrebp-*R | 5′-GAAGCCACCCTATAGCTCCC-3′ |
| *Cpt1α-*F | 5′-TGGCATCATCACTGGTGTGTT-3′ |
| *Cpt1α-*R | 5′-GTCTAGGGTCCGATTGATCTTTG-3′ |
| *Fas-*F | 5′-GGAGGTGGTGATAGCCGGTAT-3′ |
| *Fas-*R | 5′-TGGGTAATCCATAGAGCCCAG-3′ |
| *Fabp-*F | 5′-GCTGCGGCTGCTGTATGA-3′ |
| *Fabp-*R | 5′-CACCGGCCTTCTCCATGA-3′ |
| *Gapdh*-F | 5′-TGTGTCCGTCGTGGATCTGA-3′ |
| *Gapdh*-R | 5′-CCTGCTTCACCACCTTCTTGAT-3′ |
| *Pparα-F* | 5′-CTGCAGAGCAACCATCCAGAT-3′ |
| *Pparα-*R | 5′-GCCGAAGGTCCACCATTTT-3′ |
| *Scd1-*F | 5′-CCGGAGACCCCTTAGATCGA-3′ |
| *Scd1-*R | 5′-TAGCCTGTAAAAGATTTCTGCAAACC-3′ |
| *Srebp-1c-*F | 5′-GGAGCCATGGATTGCACATT-3′ |
| *Srebp-1c-*R | 5′-GGCCCGGGAAGTCACTGT-3′ |
